# Superior tunable photocatalytic properties for water splitting in two dimensional GeC/SiC van der Waals heterobilayers

**DOI:** 10.1038/s41598-021-97251-1

**Published:** 2021-09-06

**Authors:** Md. Rasidul Islam, Md. Sherajul Islam, Abu Farzan Mitul, Md. Rayid Hasan Mojumder, A. S. M. Jannatul Islam, Catherine Stampfl, Jeongwon Park

**Affiliations:** 1 Key Laboratory of Semiconductor Materials Science, Beijing Key Laboratory of Low Dimensional Semiconductor Materials and Devices, Institute of Semiconductors, Chinese Academy of Sciences, Beijing, 100083 People’s Republic of China; 2 Department of Electrical and Electronic Engineering, Green University of Bangladesh, Dhaka, 1207 Bangladesh; 3 Department of Electrical and Electronic Engineering, Khulna University of Engineering & Technology, Khulna, 9203 Bangladesh; 4 Electrical and Computer Engineering Department, Michigan State University, East Lansing, MI 48824 USA; 5 School of Physics, The University of Sydney, Sydney, NSW 2006 Australia; 6 School of Electrical Engineering and Computer Science, University of Ottawa, Ottawa, ON K1N 6N5 Canada; 7 Department of Electrical and Biomedical Engineering, University of Nevada, Reno, NV 89557 USA

**Keywords:** Energy science and technology, Engineering, Materials science, Nanoscience and technology

## Abstract

The photocatalytic characteristics of two-dimensional (2D) GeC-based van der Waals heterobilayers (vdW-HBL) are systematically investigated to determine the amount of hydrogen (H_2_) fuel generated by water splitting. We propose several vdW-HBL structures consisting of 2D-GeC and 2D-SiC with exceptional and tunable optoelectronic properties. The structures exhibit a negative interlayer binding energy and non-negative phonon frequencies, showing that the structures are dynamically stable. The electronic properties of the HBLs depend on the stacking configuration, where the HBLs exhibit direct bandgap values of 1.978 eV, 2.278 eV, and 2.686 eV. The measured absorption coefficients for the HBLs are over ~ 10^5^ cm^−1^, surpassing the prevalent conversion efficiency of optoelectronic materials. In the absence of external strain, the absorption coefficient for the HBLs reaches around 1 × 10^6^ cm^−1^. With applied strain, absorption peaks are increased to ~ 3.5 times greater in value than the unstrained HBLs. Furthermore, the HBLs exhibit dynamically controllable bandgaps via the application of biaxial strain. A decrease in the bandgap occurs for both the HBLs when applied biaxial strain changes from the compressive to tensile strain. For + 4% tensile strain, the structure I become unsuitable for photocatalytic water splitting. However, in the biaxial strain range of − 6% to + 6%, both structure II and structure III have a sufficiently higher kinetic potential for demonstrating photocatalytic water-splitting activity in the region of UV to the visible in the light spectrum. These promising properties obtained for the GeC/SiC vdW heterobilayers suggest an application of the structures could boost H_2_ fuel production via water splitting.

## Introduction

The global escalation of the consumption of carbon-based fossil fuels has increased significantly in recent years, adding increasingly more emissions of CO_2_, greenhouse gas and eventually affecting the natural climate^1–3^. It is now necessary to look for more environmentally friendly and sustainable energy alternatives to meet the energy consumption demand. Hydrogen (H_2_) based energy conversion is gaining increasing interest as a green fuel^4^. The conventional ways to produce H_2_ from water (H_2_O) involve steam methane reforming (SMR) at high temperature and pressure, biomass pyrolysis, and coal gasification (~ 5 MPa)^5^. Such a production route is often expensive and produces CO_2_ by-products. Compared with the conventional techniques of H_2_ production, solar-based water-splitting technology is environmentally safe, cost-effective, and does not produce CO_2_ by-products^6^. In the semiconductor-based photocatalysis process, solar energy is absorbed by the semiconductor material to generate electron–hole pairs (EHPs). EHPs then interact with pure water and later engender hydrogen (oxygen) from H_2_O per the evolution reaction of H_2_ (O_2_). Since 1972, the year when the first-ever water splitting was reported using TiO_2_^6,7^, a vast quantity of materials, from bulk to monolayer structures, have been considered for water splitting technology to improve photocatalysis efficiency. However, it is still a challenge to identify new, novel materials that fulfill all the requirements of photocatalysis with an enhanced efficiency.

In recent years, graphene-like two-dimensional (2D) nanostructures are being explored for photocatalysis applications with outstanding properties^8–10^. For instance, C_3_N_4_ (g-C_3_N_4_), a graphene-like metal-free 2D photocatalyst, can produce ~ 3.2 μmol/h/g of H_2_ when larger than 420 nm wavelength photons impinge upon it. The surface area of g-C_3_N_4_ is ~ 10 m^2^/g, and it exhibits a bandgap of ~ 2.7 eV in the ultra-violet (UV) region^11^. The amount of H_2_ yield (~ 3700 μmol/h/g) from the g-C_3_N_4_ is ~ 35 times higher than that obtained with Pt co-catalysts^12^. When graphene is band engineered in the presence of a co-catalyst, it generates around ~ 1050 μmol/h/g H_2_^13^. 2D transition metal dichalcogenides (TMDs) comprising MoS_2_, MoSe_2_, WS_2_, WSe_2_, CdS, ZnS, and SnS_2_, are also commonly used for photocatalysis. MoSe_2_, MoS_2_, and WS_2_ render excellent H_2_ yields that could correspondingly be as high as ~ 62,000, ~ 26,000, and ~ 2570 μmol/h/g^14,15^. 2D materials with large tunable bandgaps, including InX (X = S, Se, Te) (~ 2.22–3.19 eV) and GaX (X = S, Se, Te) (~ 2.20–2.71 eV), are also considered as photocatalysis active in the visible region^16^. The high surface area for both nanostructures introduces intensified photocatalytic activities while boosting the charge carrier’s mobility. Efficient carrier isolation, however, to prevent recombination and high bandgap tunability produces a nanostructure limiting effect. Application of doping, co-doping; introduction of defects; incorporation of external strains; employment of uniaxial or biaxial electric/magnetic fields; and construction of van der Waals heterostructures (vdWHs) are conventionally applied to reduce the limitations. Due to the characteristics of high bandgap tunability, controllable interlayer spacing, unique in-plane and interfacial thermal transport, formation of type I–II bandgap, and efficient spatial carrier separations, vdW hetero-bilayers capture the core concentration of photocatalytic operation^17–26^. In recent years, several studies focused on the advancement of photocatalytic water-splitting by forming and considering broad bandgap vdWHs^11,27–30^.

Prior investigations with vdWHs, including BP/GaN^31^, ZnO/MoS_2_^32^, GaN/GeC^33,34^, ZnO/WSe_2_^35^, blue phosphorene/g-GaN^36^, graphene/g-C_3_N_4_^37^, CdO/CdS^38^, *h*-BN/C_2_N^39^, ZnO/GeC^40^, and blue phosphorene/MoS_2_^41^, have outlined some promising optoelectronic features. These vdWHs demonstrate a highly tunable bandgap, large absorption coefficient, efficient isolation of the carrier, and contingent stacking tunability resulting in improved photocatalytic operations. While in terms of optoelectronics studies, bulk GeC has almost culminated to the saturation level, its nanostructural investigations are still in their infancy. GeC possesses high-in-plane mechanical stiffness, wide electrical bandgap, strong-electric field supporting capacities, and high thermal and chemical stabilities^42^. Its use in a vdWH could be a resourceful way to generate water-splitting properties. SiC nanostructures’ vdW features are also attractive and call for them to be deeply analyzed for photocatalytic applications. The literature regarding 2D SiC-based applications reveals several significant reports of evidence of its suitability^43^. The vdW hetero-bilayer consisting of 2D GeC and SiC could thus potentially exhibit some extraordinary photocatalytic properties. However, to the best of our knowledge, there is an absence of investigations on how 2D-GeC/SiC structures can be used to generate hydrogen (H_2_) fuel by water splitting. Usually, the intrinsic properties of 2D planar SiC: broad direct bandgap and low visible light absorption, tune the entire optoelectronic behavior of the vdWHs when exposed to light of the UV to infrared region^44^. Intensive studies thus should be expanded through engineering the intrinsic content to improve the photocatalytic and photo-absorption properties of the GeC/SiC vdW hetero-bilayer. It is also important to investigate the dependence of the optical and electronic properties of the vdWH on the alternation of the stacking pattern or incorporation of biaxial strain as ways to enhance the photocatalytic activities.

Here, we systematically investigate the photocatalytic features of 2D GeC/SiC vdW-HBL for H_2_ generation by water splitting using density functional theorem (DFT). Several vdW-HBL structures have been proposed to attain exceptional and tunable optoelectronic properties for photocatalysis applications. The dynamical stabilities of the proposed structures have been confirmed by phonon calculations. The stacking-based electronic properties and optical absorption have been calculated for all the proposed structures. We have summarized the strain-dependent optoelectronic properties of the GeC/SiC vdW hetero-bilayer and observed an indication of significant photocatalysis behavior. Our findings for the GeC/SiC vdW reveal the heterobilayer to be highly suitable for fast-performing water splitting activities.

## Methodology

The density functional theory (DFT) calculations were carried out using the Quantum Espresso^45,46^ with norm-conserving (NC) pseudopotentials^47,48^ and the Purdue Burke Ernzerhof (PBE)^49^ exchange–correlation functional. The primitive unit cell of the 2D-GeC and 2D-SiC comprises a two-atom basis, maintaining the typical valence-shell electron configurations of C [*2s*^*2*^* 2p*^*2*^], Si [*3s*^*2*^* 3p*^*2*^], and Ge [*4s*^*2*^* 4p*^*2*^]. For GeC, SiC, and the three forms of vdW-HBLs, we used a kinetic energy cut-off of 40 Rydberg (Ry) and a charge density cut-off of 350 Ry. The self-consistent function was measured with high precision by considering a convergence threshold of ~ 10^–6^ a.u. Furthermore, for atomic relaxation calculations, we set the force convergence threshold to 10^–3^ a.u. An 8 × 8 × 1 gamma-centered Monkhorst–Pack k-mesh grid was used for the first Brillouin zone integration^50^. The vacuum level was set to 20 Å to eliminate inter-image layer interactions. Since the PBE functional usually underestimates the bandgap value, we used the nonlocal Heyd-Scusena Ernzerhof (HSE-06) hybrid functional to more precisely predict the bandgap of the proposed heterostructures^51^. In addition, to include the vdW interaction between monolayers, Grimme’s DFT-D3 method was utilized^52^.

For the phonon dispersion calculations, the density functional perturbation theory (DFPT) was used^53^. The NC pseudopotential with PBE and the dynamical matrix of 4 × 4 × 4 was used to calculate the phonon characteristics. The Born and Huang system was used to separate the optical phonon modes (transverse and longitudinal)^54^. For calculation of the optical properties, we calculated the magnitude of the complex dielectric function when the HBLs were exposed to electromagnetic radiation energy (eV). The k-mesh grid is 10 × 10 × 1 for higher precision and sampling values during the calculation of the optical properties with first-order time-dependent perturbation theory^55^. The expression of the dielectric function is ε(ω) = ε_1_(ω) + jε_2_(ω), where ε_1_ and ε_2_ are the real and the imaginary parts. The imaginary portion of the dielectric function could be derived by adding the empty states as^56^,

1 $${\varepsilon }_{2}^{\alpha \beta }\left(\omega \right)=\frac{4{\pi }^{2}{e}^{2}}{\Omega }\underset{q\to \infty }{\mathrm{lim}}\frac{1}{{q}^{2}}\sum_{c, v,k}2{\omega }_{k}\delta ({\varepsilon }_{ck}-{\varepsilon }_{vk}-\omega )\times \langle {\mu }_{ck+q{e}_{\alpha }}|{\mu }_{vk}\rangle \langle {\mu }_{vk}|{\mu }_{ck+q{e}_{\beta }}\rangle .$$

Here, *c* represents the conduction band states; *ν* the valence band states; *μ*_*cκ*_ refers to the part of the wave-function that falls on the periodic cell part at k-point.

## Results and discussion

**Figure 1:**
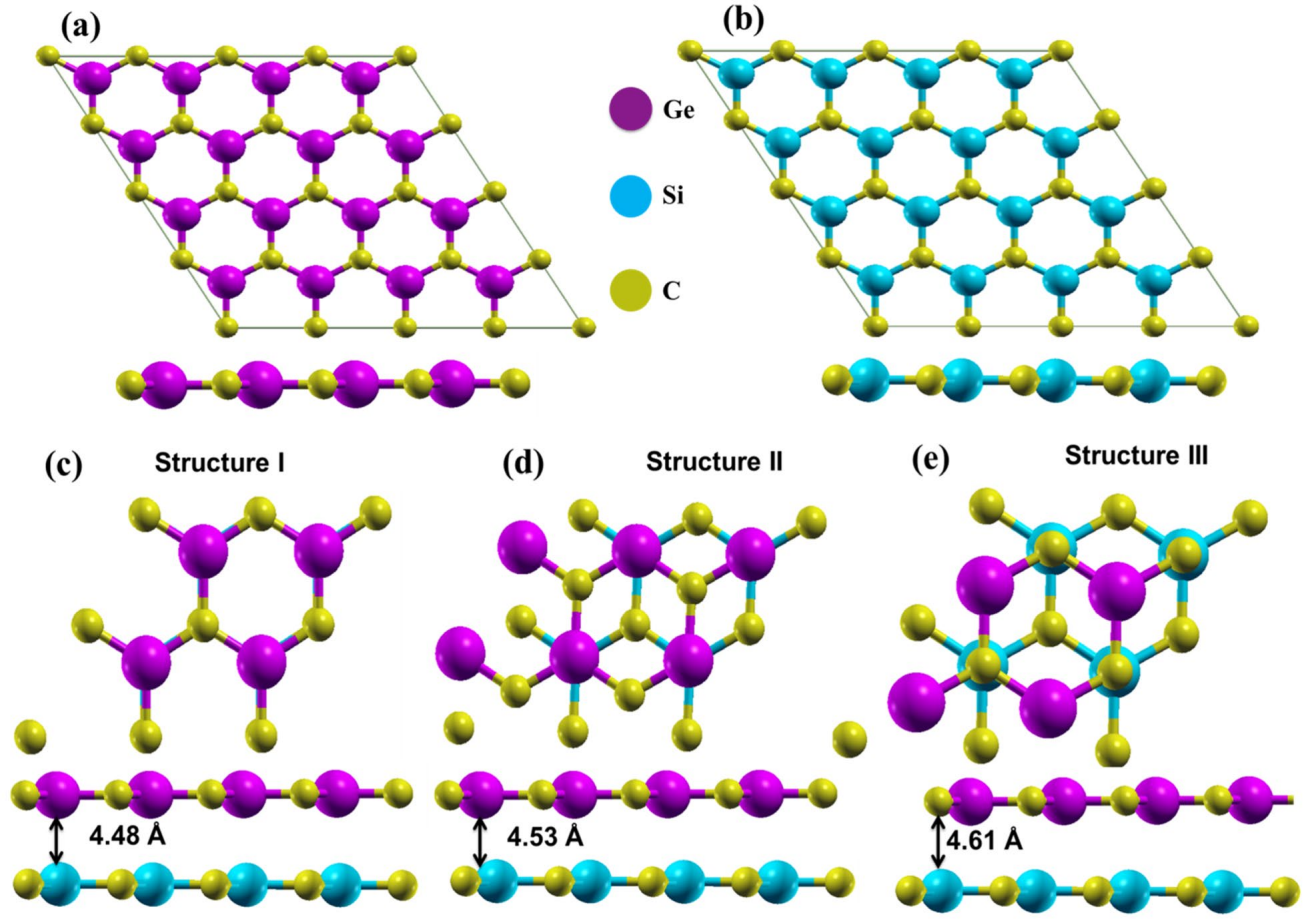
Optimized atomic structure of (**a**) 2D-GeC, (**b**) 2D-SiC, (**c**) Structure I, (**d**) Structure II, and (**e**) Structure III. Golden yellow, violet, and cyan spheres represent C, Ge, and Si, respectively.

The structural properties of the GeC/SiC vdW-HBL materials are calculated systematically. The optimized structures of the monolayer GeC, SiC, and three types of vdW-HBLs are shown in Fig. 1. The 2D-GeC and 2D-SiC are planar graphene-like buckling-free honeycomb structures. Here, for the geometrically relaxed planar GeC monolayer, we calculated the bond length to be 1.875 Å and lattice constant, $a$ = 3.246 Å. We obtained 1.847 Å for the bond length and 3.138 Å for the lattice constant for the SiC monolayer. These findings match closely with the previous results^21,57,58^. We considered three different stacking patterns by placing a 2 × 2 2D-GeC ($a$ = $b$ = 6.276 Å) layer above a 2 × 2 2D-SiC ($a$ = $b$ = 6.492 Å) monolayer with different configurations.

The lattice constants between the GeC and SiC are closely matched, and they maintain similar crystal structures. The lattice mismatch between GeC and SiC layers is calculated using the relation, $f=\frac{{a}_{GeC}-{a}_{SiC}}{{a}_{GeC}}\times 100\%$, where, ${a}_{GeC}$ and ${a}_{SiC}$ represent the lattice constant of GeC and SiC, respectively. The lattice mismatch is approximately 3.3% between GeC and SiC, which is considerable for a vdW-HBL. Among the three considered stacking patterns of the HBL: in structure I, the Ge and C atoms of the GeC monolayer are placed directly above Si and C of the lower SiC monolayer; in structure II (III), Ge (C) atoms were placed at the direct vertical line above the lower Si atoms, but the C (Ge) atoms were fixed at the center of the hexagons.

**Figure 2:**
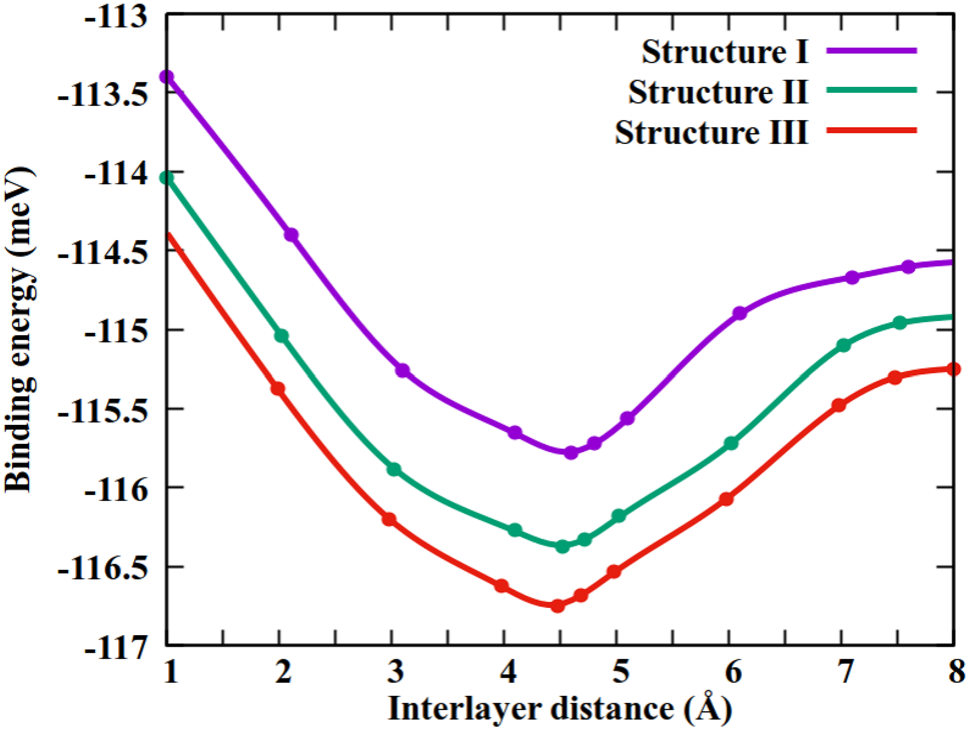
Binding energy versus interlayer distances for the three vdW heterostructures.

The structures I-III, after dynamic stability analysis and being geometrically-relaxed, show lattice constants of ~ 3.221 Å, ~ 3.223 Å, and ~ 3.226 Å, respectively. The interlayer binding energy during the relaxation process is obtained using the following formula,

2 $${E}_{b}={E}_{GeC/SiC}-{E}_{GeC}-{E}_{SiC},$$

where, ${E}_{GeC/SiC}$ refers to the total energy of the HBL (including dispersion correction), ${E}_{GeC}$ and ${E}_{SiC}$, are the total energies of the 2D-GeC and 2D-SiC monolayers, respectively. The change of binding energy as a function of interlayer distance is demonstrated in Fig. 2 for the three vdW-HBLs. The geometry-relaxed lattice constants, interlayer binding energy, corresponding interlayer spacing, and bond lengths are listed in Table 1 for 2D-GeC, 2D-SiC, and the HBL structures I–III. It can be seen that structure III is the most energetically favorable.

**Table 1:** The geometry-relaxed lattice constant *a* (Å), bond length *d*_*B*_ (Å), interlayer binding energy *E*_*b*_ (meV), interlayer spacing *d* (Å), the computed bandgap using the PBE and HSE-06 functional are *E*_g_ (PBE) (eV) and *E*_g_ (HSE-06) (eV), respectively.

Structure	*a* (Å)	*d*_*B*_ (Å)	*E*_*b*_ (meV)	*d* (Å)	*E*_*g*_ (PBE)	*E*_*g*_ (HSE-06)
2D-GeC	3.246	1.874	–	–	2.08	2.843
2D-SiC	3.138	1.794	–	–	2.52	3.234
Structure-I	3.243	–	− 115.76	4.48	1.284	1.978
Structure-II	3.254	–	− 116.35	4.53	1.486	2.278
Structure-III	3.255	–	− 116.78	4.61	1.828	2.686

**Figure 3:**
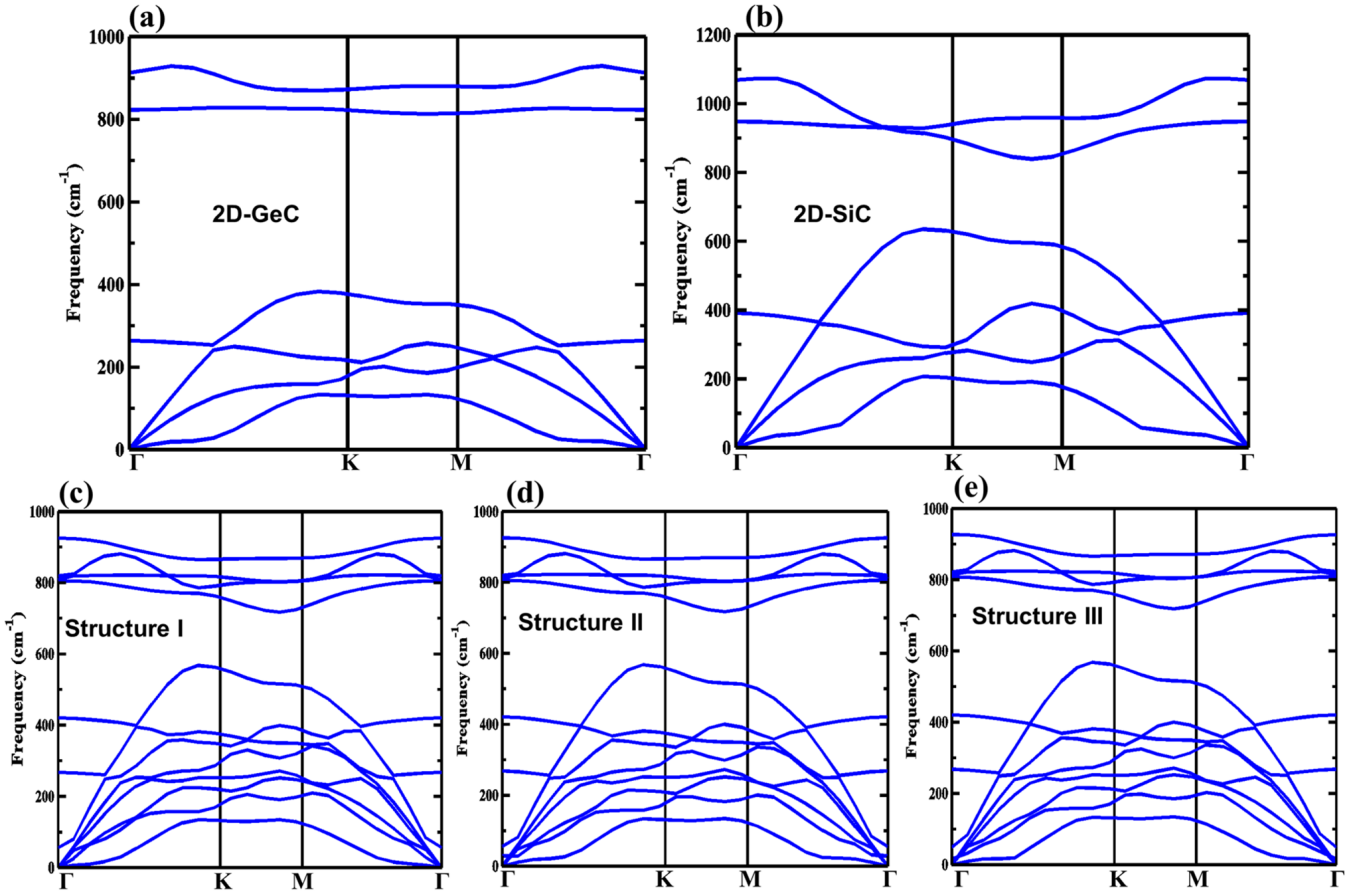
The phonon band dispersions for (**a**) 2D-GeC, (**b**) 2D-SiC, (**c**) Structure I, (**d**) Structure II, and (**e**) Structure III along the high symmetry points of first Brillouin zone.

The dynamical stability of the proposed structures is considered by calculating the phonon dispersion curves using DFPT. Due to the lack of imaginary branches in the phonon spectra, as is depicted in Fig. 3, structures I–III are dynamically stable. The three acoustic modes are the longitudinal acoustic (LA), transverse acoustic (TA), and flexural or out-of-plane acoustic (ZA) modes. These frequency modes have the lowest value at the Г point. The longitudinal optical (LO), out-of-plane optical (ZO), and transverse optical (TO) modes are the higher frequency modes. The optical phonon modes LO and TO are non-degenerate at Г for the vdW-HBLs. The highest frequency of the LO phonon mode is 921 cm^−1^, 923 cm^−1^, and 925 cm^−1^ for structure I, structure II, and structure III, respectively. For all the considered vdW-HBLs, a considerable phonon bandgap was found among the optical phonons and acoustic phonons at the K-points of the first Brillouin zone. Since all the vdW-HBLs are created by integrating two binary atomic systems, the constituent atoms of each system have a large mass difference, which causes the phonon bandgap.

**Figure 4:**
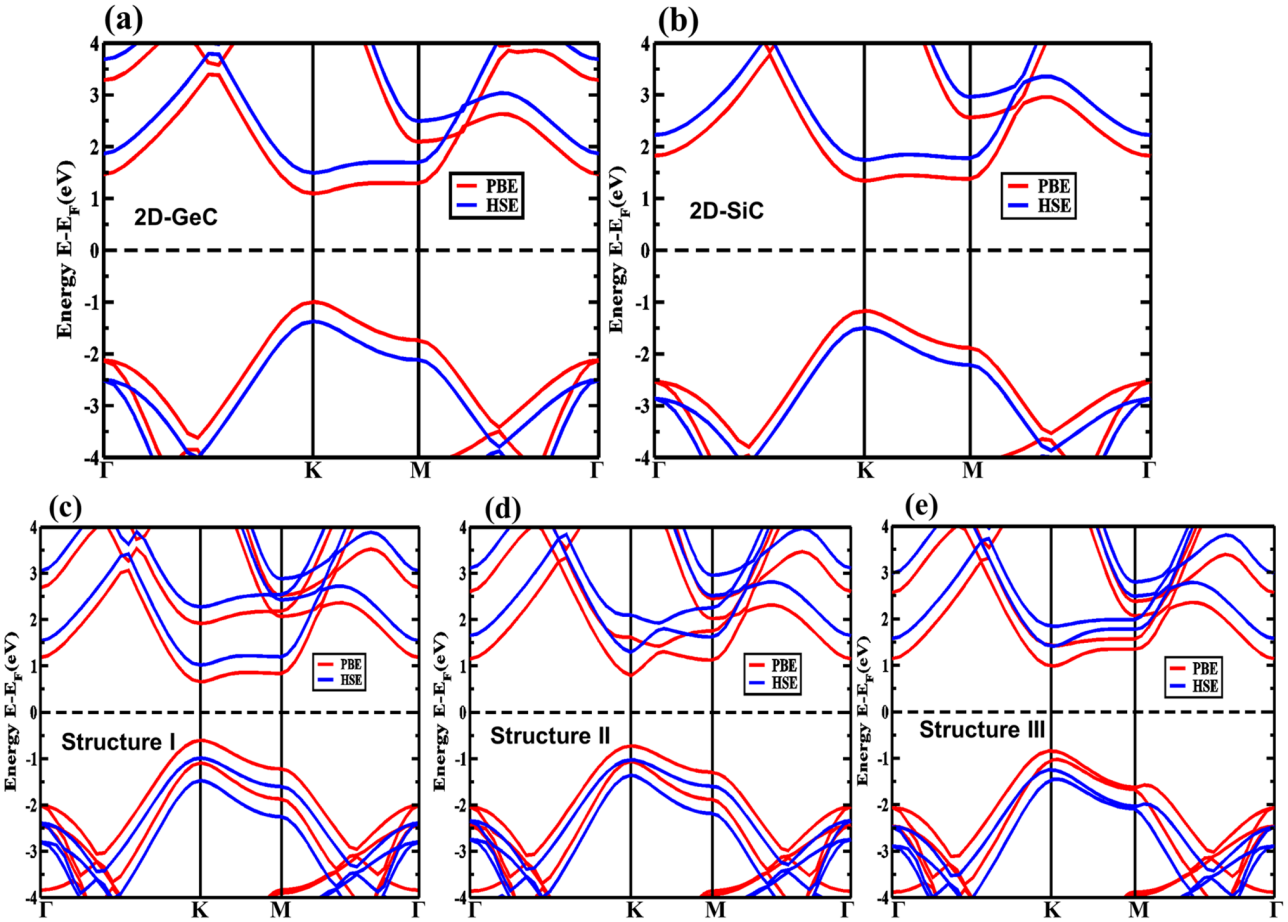
The electronic band structures for (**a**) GeC monolayer (2D-GeC), (**b**) SiC monolayer (2D-SiC) and for the vdW heterostructures. (**c**) Structure I, (**d**) Structure II, and (**e**) Structure III using the PBE and HSE-06 functionals.

We now consider the electronic properties of the 2D-GeC, 2D-SiC, and three vdW-HBLs structures. The GeC and SiC monolayer band structures are first considered to observe the effect of the PBE and HSE-06 functionals on the electronic band structure. In Fig. 4, the calculated band structures using the PBE and HSE-06 functionals are shown in red and blue lines, respectively. The observed direct energy bandgap value of the 2D-GeC and 2D-SiC structures are 2.843 eV and 3.234 eV, respectively, using the HSE-06. It is found that the bandgap is larger with the HSE-06 than PBE as usual. The bandgap value of GeC (Fig. 4a) and SiC (Fig. 4b) agrees closely with the previous theoretical studies^58,59^. The band structures of the three vdW-HBLs (with PBE and HSE06) are shown in Fig. 4c–e. It can be seen from the GeC monolayer (Fig. 4a), SiC monolayer (Fig. 4b), and the three vdW-HBLs (Fig. 4c–e) that the conduction band minimum (CBM) and valence band maximum (VBM) are located at the K-point; thus, the system is a direct bandgap structure. For an application like the photocatalysis process, a broad direct bandgap is an advantage and desirable. The bandgap values of the HBL structures I-III are 1.978 eV, 2.278 eV, and 2.686 eV, respectively. The bandgap values obtained with the PBE and HSE-06 functionals for all the structures are also summarized in Table 1. We set the center of the bandgap to zero eV in Fig. 4. The location of the band-edge is of high importance to demonstrating photocatalytic water splitting actions. We consider the HSE-06 bandgaps to explore the band edges using the equations,

3 $${E}_{CBE}=X-\frac{{E}_{g}^{HSE}}{2},$$

4 $${E}_{VBE}=X+\frac{{E}_{g}^{HSE}}{2}.$$

The *X* denotes the geometric mean of the Mulliken electro-negativities, considering all the atoms of the HBLs. We obtained an *X* for GeC of 5.25 eV, and for the SiC monolayer of 5.47 eV. Moreover, Mulliken electro-negativities for C, Si, and Ge atoms are 3.83 eV, 2.56 eV, and 2.09 eV, respectively. Then, the measurement is expanded on to the band edge for all the proposed GeC/SiC HBLs; and the water-splitting photocatalytic H_2_ production is investigated.

Generally, some criteria need to be met for photocatalytic H_2_ production by the water-splitting process in semiconductors^34^: (i) The energy of the conduction band edge (CBE) must be set to a value greater or equal to − 4.44 eV from the vacuum (reduction potential) for creating hydrogen by a reduction reaction (H^+^/H_2_), (ii) The energy of the valence band edge (VBE) must be set to a value lower or at least equal to − 5.67 eV from the vacuum (oxidation potential) to produce oxygen by oxidation reaction of water (O_2_/H_2_O), (iii) The semiconductor material must maintain a bandgap of at least ~ 1.23 eV, (iv) In the near-ultraviolet (UV) and visible solar spectrum, it must have prominent absorption peaks to utilize much of the energy spectrum, and (v) The materials should preserve a broad surface to volume ratio to enhance the photocatalysis process and to promote H_2_ formation.

**Figure 5:**
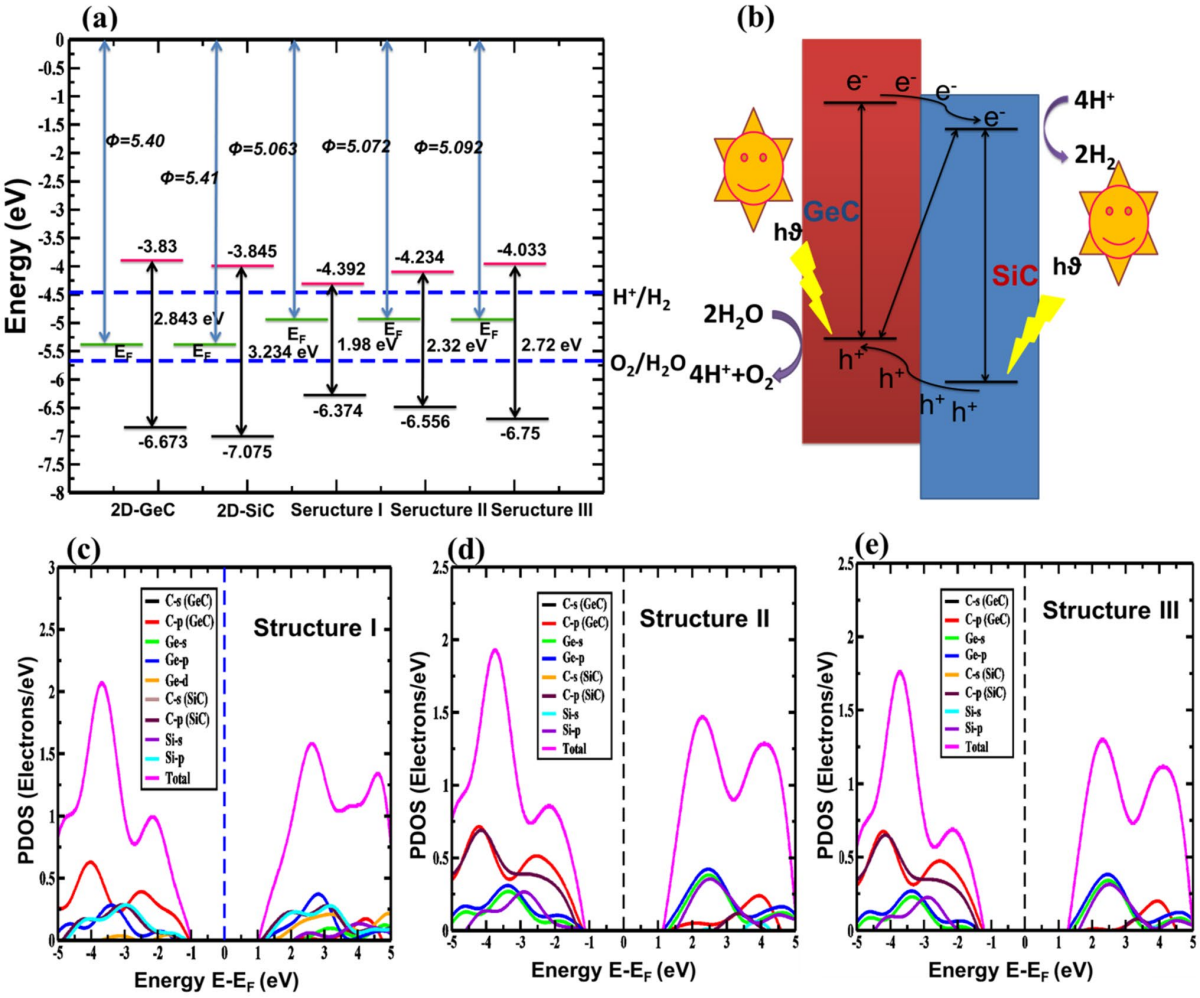
(**a**) The relative band alignment for 2D-GeC, 2D-SiC, Structure I, Structure II, and Structure III for photocatalytic water splitting. (**b**) Carrier transfer and separation mechanism in the type-II GeC/SiC vdW heterostructure. Atomic orbital projected density of states (PDOS) for (**c**) Structure I, (**d**) Structure II, and (**e**) Structure II vdW heterostructures using the HSE-06.

The band edge locations of GeC, SiC, and the considered GeC/SiC vdW-HBLs are shown in Fig. 5a. The location of the valence band maximum (VBM) and the conduction band minimum (CBM) refers to a type-II band structure for the HBLs. We observed the energy level at which the reduction process (2H^+^/H_2_) and oxidation process (H_2_O/O_2_) occurs being − 4.46 eV and − 5.67 eV, respectively. Also, the energy associated with the conduction (valence) band edge is much greater (lower) than the potential of oxidation (reduction). Thus, the HBL structures will show sufficient over-potential, so much as to cause photocatalysis. Additionally, we ensured the operative carrier parting during the photocatalysis process by individually measuring the valence band offset (0.402 eV) and conduction band offset (0.015 eV) value. The photocatalytic mechanism of the proposed GeC/SiC heterostructure is demonstrated in Fig. 5b. It can be seen that both the valence and conduction bands of the SiC layer lie below that of the GeC layer, forming a type-II heterostructure^60^. Thus, when light strikes this type-II GeC/SiC heterostructure, the photogenerated electron from the conduction band of GeC could easily get excited to surpass the conduction offset between the GeC and SiC bands, causing the electron to drift towards the conduction band of the SiC. Similarly, the holes from the valence band of the SiC layer will move onto the valence band of the GeC layer, as depicted in Fig. 5b. Such a redistribution of the photogenerated charge carriers actually promotes water splitting characteristics. The electron that reached the conduction band of the SiC could be used in redox reactions to form H_2_ molecules from the H^+^ charge. At the same time, the valence band of the GeC layer will enhance oxidation reaction by dissipating H_2_O molecules into H^+^ charge and O_2_ atoms. Moreover, among the three considered structures, structure-III is predicted to show the most photocatalytic activity due to the higher separation between the band edge locations.

The complete and partial density of states was calculated to determine the orbital contribution to the formation of the total density of states (Fig. 5c–e). It is found that in structure I, the sp^2^ hybridized p-orbital of Ge principally contributes to the CBM. In contrast, the VBM is formed primarily by the sp^2^ hybridized p-orbital of C atoms of the GeC monolayer. As a result, structure I shows a form-I straddling band configuration. In structure II and structure III, the p-orbital of C and Si atoms of the GeC and SiC layer mainly contributes to the formation of the VBM and CBM, respectively, and results in a form-II staggered orientation of the band.

We now consider the work function of the GeC/SiC HBLs. The work function is defined as, $\Phi ={E}_{vacuum}-{E}_{Fermi}$; the amount of energy required to move an electron from the uppermost occupied state to the vacuum. The calculated work function for the GeC monolayer is 5.40 eV, and for the SiC, it is 5.41 eV. It can be concluded that before obtaining the equilibrium Fermi stage, there is an unswerving of the electron from SiC to GeC monolayer. The calculated work function values for the HBL structures I–III are 5.063 eV, 5.072 eV, and 5.092 eV. Thus, the variation of the structural configuration of the HBLs does not lead to any significant alteration of the work function.

**Figure 6:**
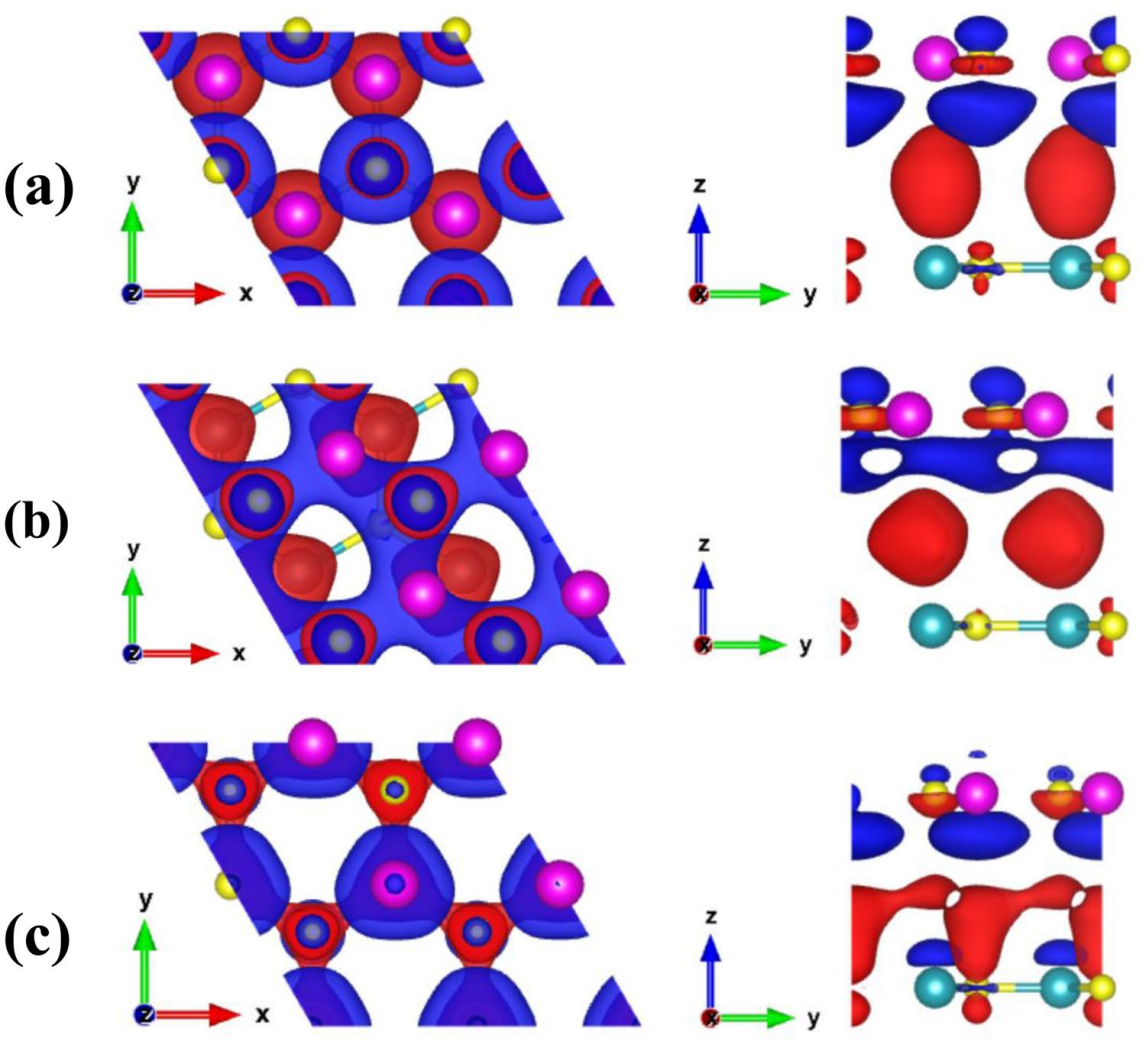
Charge density difference (CDD) between the top (GeC) and bottom (SiC) layer of the heterobilayers: (**a**) Structure-I, (**b**) Structure-II, and (**c**) Structure-III. An isosurface value of 0.00057 ${e\AA }^{-3}$ was used. Left: top view, right: side view. The red and blue color refers to the depletion and accumulation of charge, respectively. Golden yellow, violet, and cyan spheres represent C, Ge, and Si, respectively.

The charge density difference (CDD) for the three stacking configurations was calculated to determine the atomic contribution to the charge transfer between the GeC and SiC monolayers. In Fig. 6, the CDD for the three structures is shown, as calculated using the relation, $\Delta {\rho }_{CDD}={\rho }_{HBL}-{\rho }_{GeC}-{\rho }_{SiC}$; where, $\Delta {\rho }_{CDD}$ is the CDD; ${\rho }_{HBL}$, ${\rho }_{GeC}$, and ${\rho }_{SiC},$ refer to the charge density (CD) of the considered HBL structure, the GeC monolayer, and the SiC monolayer, respectively. The charge accumulation (depletion) around the atomic surfaces is marked by the blue (red) color. For structure I (Fig. 6a) and structure II (Fig. 6b), the charge depletes from the Ge atoms and accumulates near the C atoms of the GeC layer. A small amount of charge also depletes near the C atoms of the SiC layer. However, in structure III (Fig. 6c), most of the charge depletes from the C atom of the bottom SiC layer, and an accumulation occurs in the proximity of the Ge atom of the top GeC layer. For all the three heterobilayer structures, in general, charge accumulates near the GeC layer and depletes near the SiC layer, resulting in an effective charge transfer from the SiC to the GeC layer. Such a charge transfer character indicates that GeC will largely determine the properties of the considered heterobilayers; it will afford most electron transport through the GeC layer, causing significantly higher tunable bandgap values for the heterobilayer. And, the SiC layer acts as a “property modulator” of the heterobilayers. The surface electrostatic potential is important to predict the amount of positive and negative charges near the active sites at the surface. The calculated electrostatic potential for all the considered structures is represented in Supplementary Fig. S1. Two negative peaks are found, with the potential at the GeC layer being substantially deeper than that of the GeC layer, suggesting that a charge shift from the SiC to the GeC monolayer may occur. It can also be observed that the structures differ very little in terms of layer projected surface electrostatic potential value.

The charge distribution at the CBM and VBM of the considered heterobilayers is depicted in Supplementary Fig. S2. The charge density at the CBM and VBM is concentrated mostly at the Ge atoms of the GeC layer and the C atoms of the SiC layer, respectively. Thus, an efficient separation between the electron at the GeC layer and the hole at the SiC layer is obtained. Increased carrier pair separation reduces carrier recombination rate and improves solar energy utilization. The fabrication of 2D exciton solar cells or the development of photocatalytic water splitting solar energy collecting devices are frequently possible due to this phenomenon. The efficacy of the water-splitting process can be defined by the number of electrons and holes at the conduction and valence bands active sites, respectively. From the charge distribution at the VBM and CBM, the electron and holes accumulated near the Ge and C atoms of the GeC and SiC layer will supply adequate reaction sites to carry out the oxidation and deoxidation reactions. Thus, H_2_ fuel can be produced by utilizing the GeC/SiC heterobilayer as a photocatalyst.

**Figure 7:**
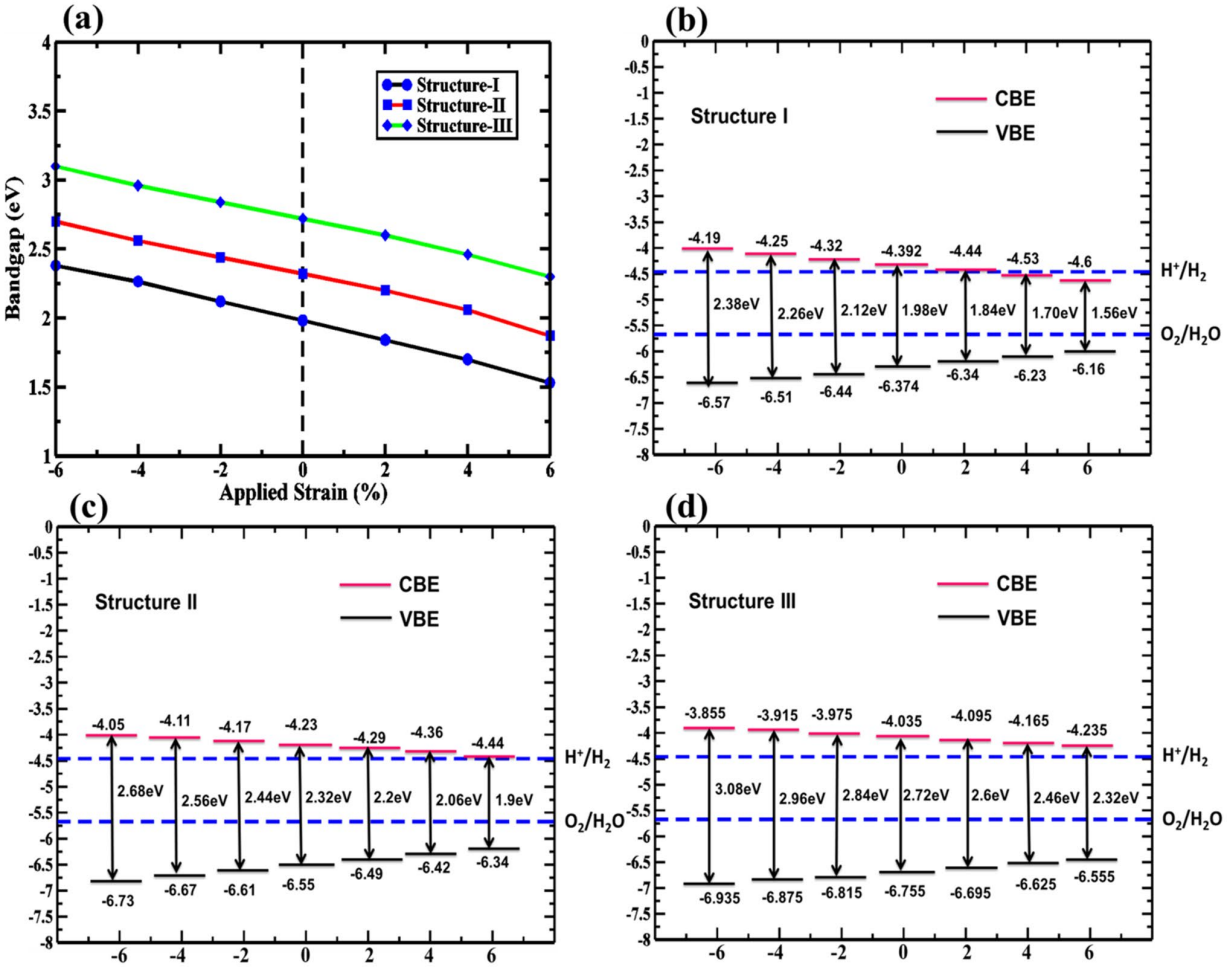
Strain-dependent (**a**) bandgap and relative band edge position for (**b**) Structure I, (**c**) Structure II, and (**d**) Structure III vdW heterostructures using the HSE-06 functional.

Biaxial strain has a significant impact on the electronic characteristics of heterostructures. We, therefore, incorporated biaxial strains into the HBLs to investigate the degree of modification of the electronic characteristics as induced by the external strain. Here, the biaxial strain in the range of − 6% to + 6%, at a 2% increment, is considered. In Fig. 7a, the change in bandgap with applied biaxial strain is shown. From Fig. 7, it can be seen that the biaxial strain results in a noticeable reduction of the bandgaps. When the strain is changed from − 6% to + 6%, the CBM and VBM move toward the Fermi level. It was found that for both the PBE and HSE-06 exchange–correlation functions, the bandgap values are always reduced when going from the compressive to the tensile strain. Throughout the change of the biaxial strain, the bandgap remains direct. Due to the lowering of the bandgap values, the absorption range for the HBLs under strain also extends from the UV to the visible range and demonstrates photocatalytic activities. Therefore, the tunability of electronic properties via strain is a promising approach to enhance the water-splitting activities of the hetero-bilayer systems. The charge redistribution in VBM and CBM upon application of biaxial strain is also shown in Supplementary Fig. S3.

The conduction and valence band edges and oxidation and reduction potential of water are also considered to calculate the amount of H_2_ production by absorbing electromagnetic radiation. For strain-induced structure I, structure II, and structure III, we have therefore determined the relative band edge position, as seen in Fig. 7b–d. For structure I, at + 4% tensile strains, the conduction band energy is lower than the energy required for the reduction process of water. Structure II and structure III, however, have sufficient kinetic over-potential within the range of − 6 to + 6% biaxial strains. Our findings indicate that the obtained kinetic overpotential, resulting from the tailorable electronic features by changing the applied biaxial strain, makes the HBL systems promising for photocatalytic water division.

**Figure 8:**
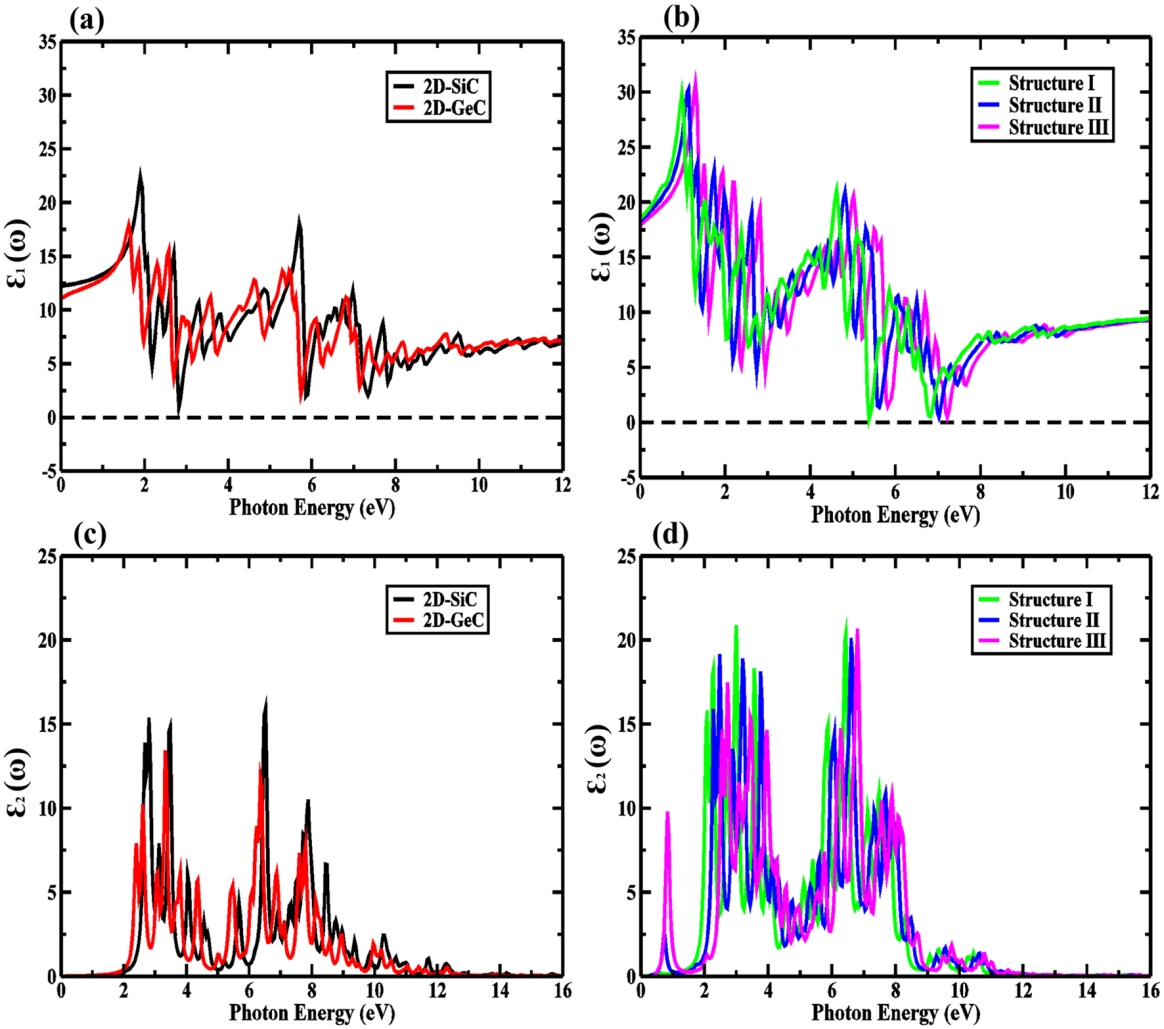
The real and imaginary parts of the dielectric constant for (**a**,**c**) 2D-GeC and 2D-SiC, (**b**,**d**) real and imaginary dielectric constant for Structure I, Structure II, and Structure III.

The optical properties of the proposed vdW-HBL structures were investigated with the first-order DFPT. The two parts of the complex dielectric constant: the real and the imaginary, represent the effect of dispersion and absorption. For obtaining the real part of the dielectric constant, we incorporated the Kramers–Kronig transformation, whereas the imaginary part was calculated through the components of the momentum matrix. The transport of charge carriers from the valence to the conduction band depends on the peaks of the imaginary dielectric component. The complex dielectric function, represented by $\varepsilon (\omega ) = {\varepsilon }_{1}(\omega ) + i{\varepsilon }_{2}(\omega )$, is the expression for the response of semiconductor materials when any energy spectrum with angular frequency (ω) strikes it. According to the underlying physics of dielectric materials, the complex dielectric function is highly susceptible to absorption loss (dispersion effect). The absorption coefficient of the materials can be expressed through the real and imaginary dielectric functions parts as,

5 $$\alpha \left(\omega \right)=\sqrt{2}{(\sqrt{{\varepsilon }_{1}^{2}\left(\omega \right)+{\varepsilon }_{2}^{2}\left(\omega \right)}-{\varepsilon }_{1}\left(\omega \right))}^{1/2},$$

where *ω* denotes the photon frequency, and *α* represents the absorption coefficient. The calculated real and imaginary parts of the dielectric function for 2D-GeC, 2D-SiC, and the three proposed HBLs are shown in Fig. 8. For structure II and structure III, there is an absence of negative part of the real dielectric function ${\varepsilon }_{1}(\omega )$. For structure I, ${\varepsilon }_{1}(\omega )$ is negative between ~ 5 eV to 6 eV. Therefore, structure I displays the most metallic activity. Due to this, the HBLs could be considered for an application like nano-coating of substances in the ultra-violet (UV) regime. Moreover, due to bandgap tailoring, radiation near to the visible to UV range shows an improved refractive index with positive ε $(\omega )$. As shown in Fig. 8b, the lower end of the absorption peaks in ${\varepsilon }_{2}(\omega )$, are at 2.9 eV, 3 eV, and 3.1 eV for structures I–III, respectively, and corroborate the bandgaps obtained under DFT.

**Figure 9:**
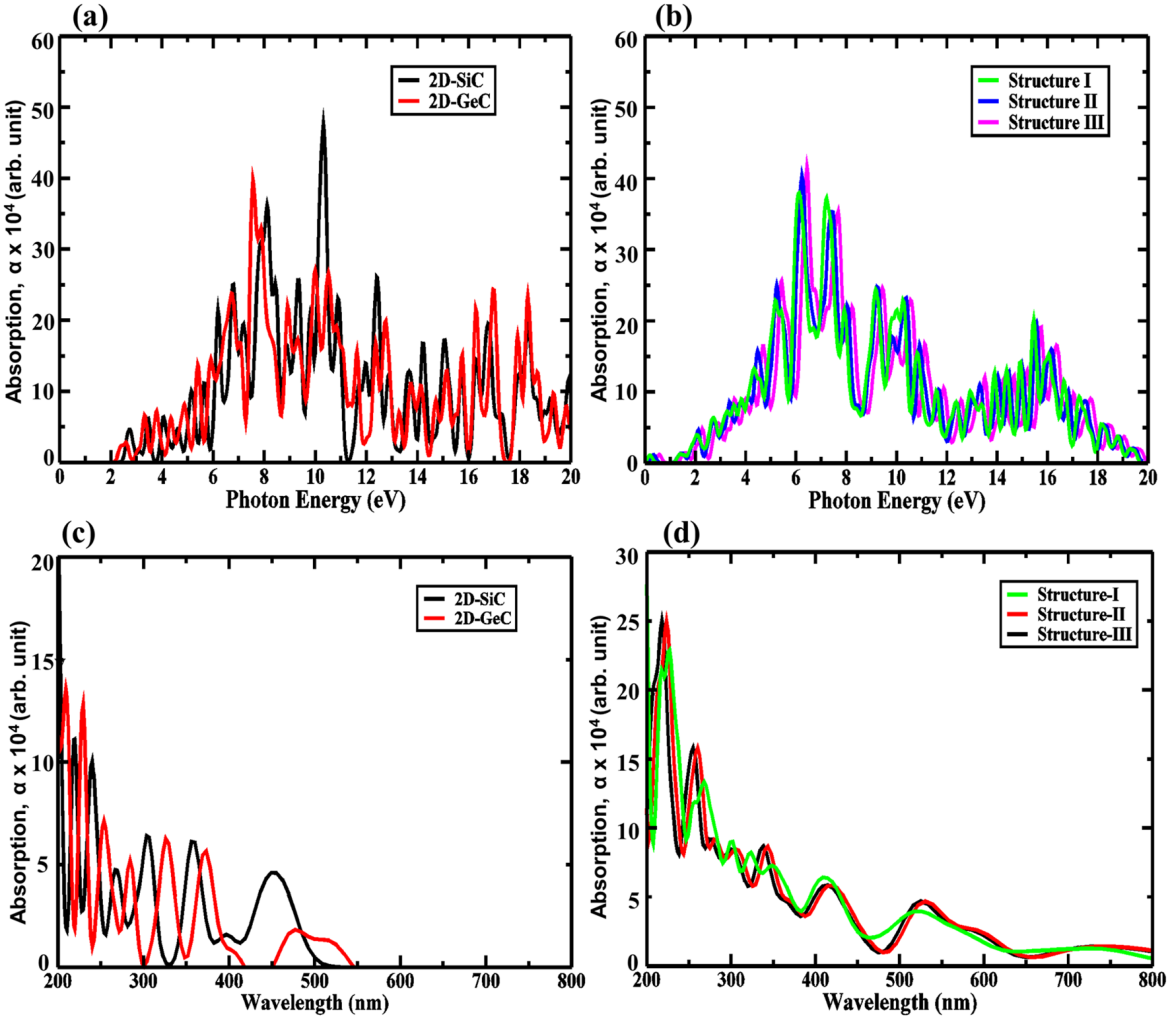
Absorption properties for (**a**,**c**) 2D-GeC and 2D-SiC, (**b**,**d**) for Structure I, Structure II, and Structure III.

From Fig. 9, it can be seen that compared to the monolayers of GeC and SiC, our considered GeC/SiC HBLs exhibits higher absorption levels. The maximum absorption obtained is 3.9 × 10^5^ cm^−1^, 4 × 10^5^ cm^−1^, and 4.1 × 10^5^ cm^−1^ for structures I–III, respectively, at 6.4 eV, 6.5 eV, and 6.6 eV, which falls into the practical application range of optoelectronic devices^45^. High intensity and a larger number of absorption points cause higher photocatalysis and the generation of electron–hole pairs. Moreover, the larger concentration of peaks infers that more carrier transitions and greater electron mobility are also present for structures I–III. We obtained the absorption coefficient value near the visible region (~ 3.4 eV) can be as large as ~ 1 × 10^5^ cm^−1^.

**Figure 10:**
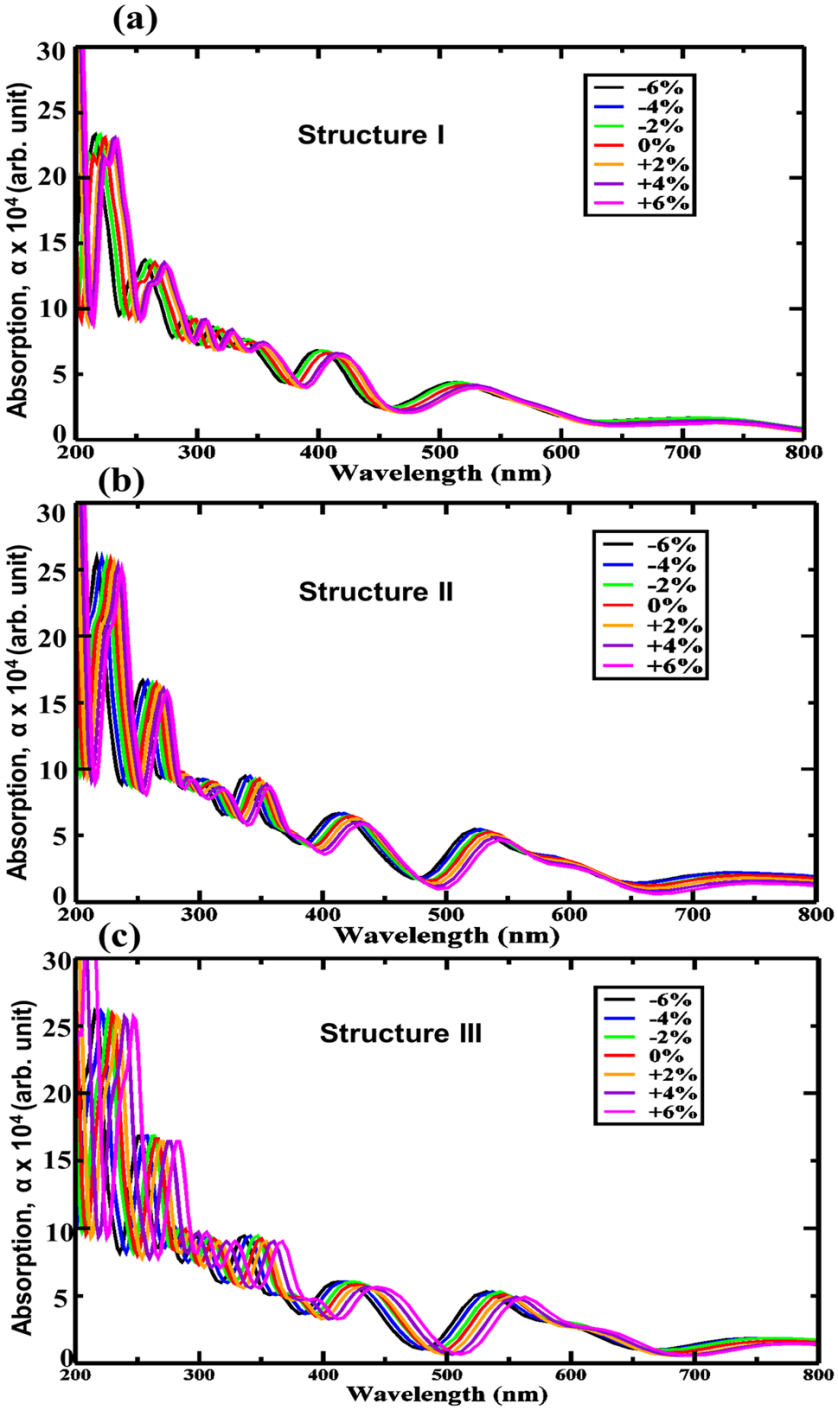
Strain-induced absorption properties for (**a**) Structure I, (**b**) Structure II, and (**c**) Structure III.

In perovskite materials, absorption of light from the UV to IR range can show a photocatalysis effect in HBLs^61^. The shift of absorption peaks can be achieved by applying biaxial strain, which alters the absorption coefficient and renders more efficient utilization of the solar spectra. In this context, for the GeC/SiC HBLs, we considered the optical features under the influence of varying biaxial strain. Figure 10a–c show the absorption profile of structures I–III, respectively, while the strain is varied from − 6% to + 6%. The absorption peaks appear at ~ 410 nm, ~ 415 nm, and ~ 430 nm wavelength for the three structures, respectively, and results in an absorption coefficient of ~ 7 × 10^5^ cm^−1^, ~ 7.2 × 10^5^ cm^−1^, and ~ 7.3 × 10^5^ cm^−1^. The absorption peaks, however, shift to the UV region with progressively higher compressive strain. It can be observed that structure III exhibits the most sensitivity to the applied strain. More peaks appear near the lower UV region. The bandgap values reduce with the applied biaxial strain, and while doing so, shifts the absorption peaks. The presence of absorption peaks in the UV and visible light spectrum initiates the opportunity to use a wider range of energies for the water-splitting process.

## Conclusions

In this investigation, the feasibility of photocatalysis yielding H_2_ from water is analyzed for proposed novel 2D GeC/SiC vdW heterobilayers using first-principles density functional theorem calculations. Calculation of the phonon dispersion curves shows that the considered three heterobilayers of GeC/SiC are dynamically stable. Of the three HBLs, the calculation of binding energy indicates structure-III is structurally and chemically more favorable than the other two. The calculated bandgaps are 1.978 eV, 2.278 eV, and 2.686 eV for the heterobilayer structures I–III, respectively, as obtained with the HSE-06. Application of compressive and tensile biaxial strain tailors the bandgap values of the HBLs and modifies the band edge potential value. This, in turn, increases the ability of the structures to be active over a more extended range of electromagnetic radiation, especially the near-visible and UV range. The structures show a monotonic decrease in the bandgap when strain is changed from more compressive to more tensile. In the heterobilayer structures, the obtained effective mass of the electron surpasses the effective mass of the electron of the GeC and SiC monolayers and renders very high carrier mobility. Without any strain, the absorption coefficient for the HBLs could be as high as 1 × 10^6^ cm^−1^ and presides primarily near the UV range. With the incorporation of external strain, the absorption peaks could reach around ~ 3.5 times greater value than the unstrained HBLs. Our findings indicate that the 2D GeC/SiC HBLs could be used for fast-responsive photocatalytic water-splitting to produce H_2_ fuel.

## Supplementary Information

Supplementary Figures.
