# Supplementary material for: Superior tunable photocatalytic properties for water splitting in two dimensional GeC/SiC van der Waals heterobilayers
Source: Sci Rep. 2021 Sep 6;11:17739. doi: 10.1038/s41598-021-97251-1 (PMC8421365; doi:10.1038/s41598-021-97251-1)
Supplement: Supplementary file 1 — Supplementary Figures. [file 41598_2021_97251_MOESM1_ESM.docx]

**Superior** **tunable photocatalytic properties for water splitting in two dimensional GeC/SiC van der Waals heterobilayers**

Md. Rasidul Islam^a,b^, Md. Sherajul Islam*^c,g^, Abu Farzan Mitul ^d^, Md. Rayid Hasan Mojumder^c^, A.S.M. Jannatul Islam^c^, Catherine Stampfl^e^, Jeongwon Park^f,g^

^a.^Key Laboratory of Semiconductor Materials Science, Beijing Key Laboratory of Low Dimensional Semiconductor Materials and Devices, Institute of Semiconductors, Chinese Academy of Sciences, Beijing, 100083, P. R. China.

^b^Department of Electrical and Electronic Engineering, Green University of Bangladesh, Dhaka 1207, Bangladesh.

^c^Department of Electrical and Electronic Engineering, Khulna University of Engineering & Technology, Khulna-9203, Bangladesh.

^d^Electrical and Computer Engineering Department, Michigan State University, MI 48824, USA

^e^School of Physics, The University of Sydney, New South Wales 2006, Australia

^f^School of Electrical Engineering and Computer Science, University of Ottawa, Ottawa,ON K1N 6N5, Canada

^g^Department of Electrical and Biomedical Engineering, University of Nevada, Reno, NV 89557, USA

*Corresponding Author’s E-mail address: [sheraj_kuet@eee.kuet.ac.bd](mailto:sheraj_kuet@eee.kuet.ac.bd)

**
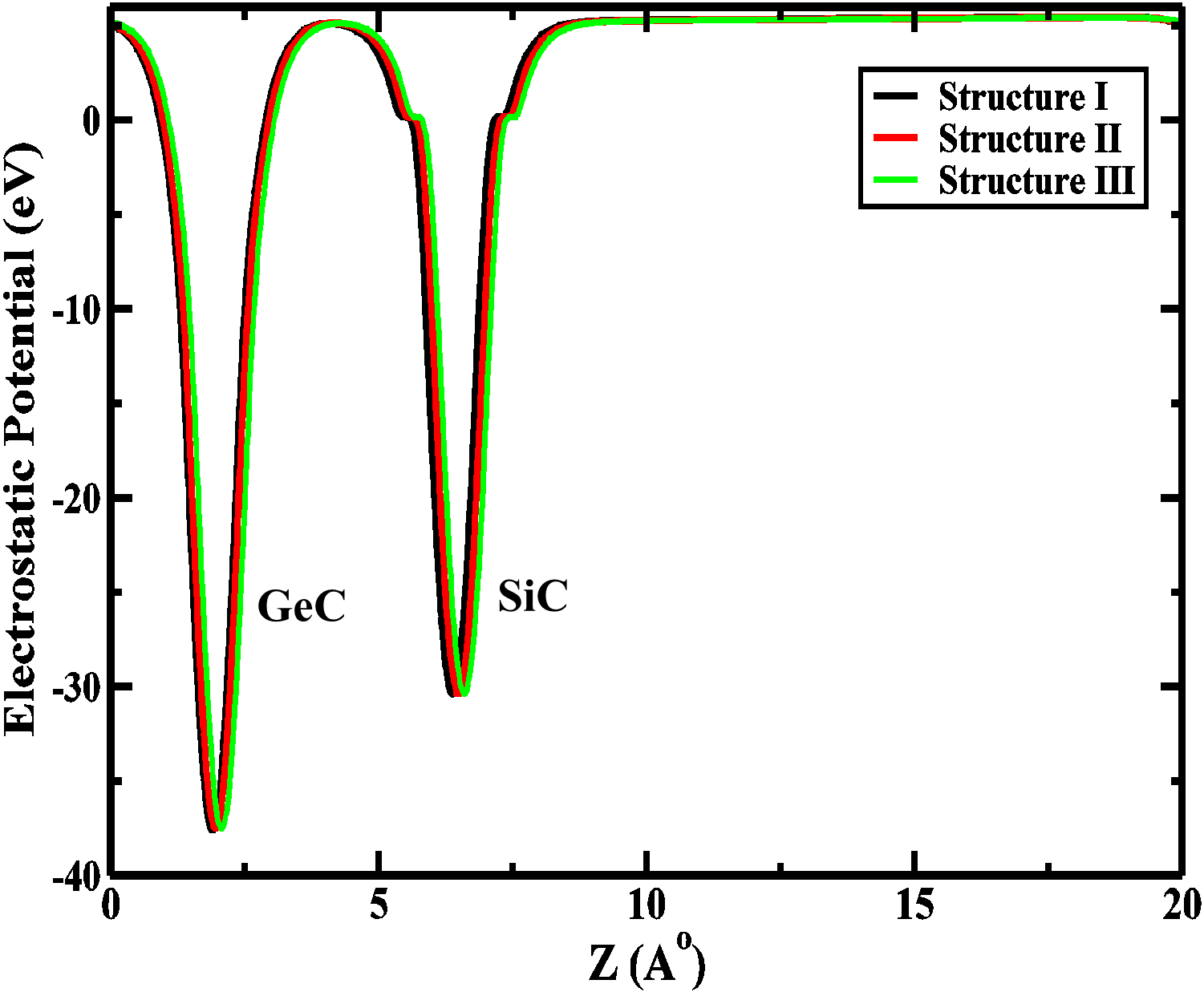
**

**Figure S1**. Electrostatic potential of three vdW-HBLs structures at the equilibrium configuration.

**
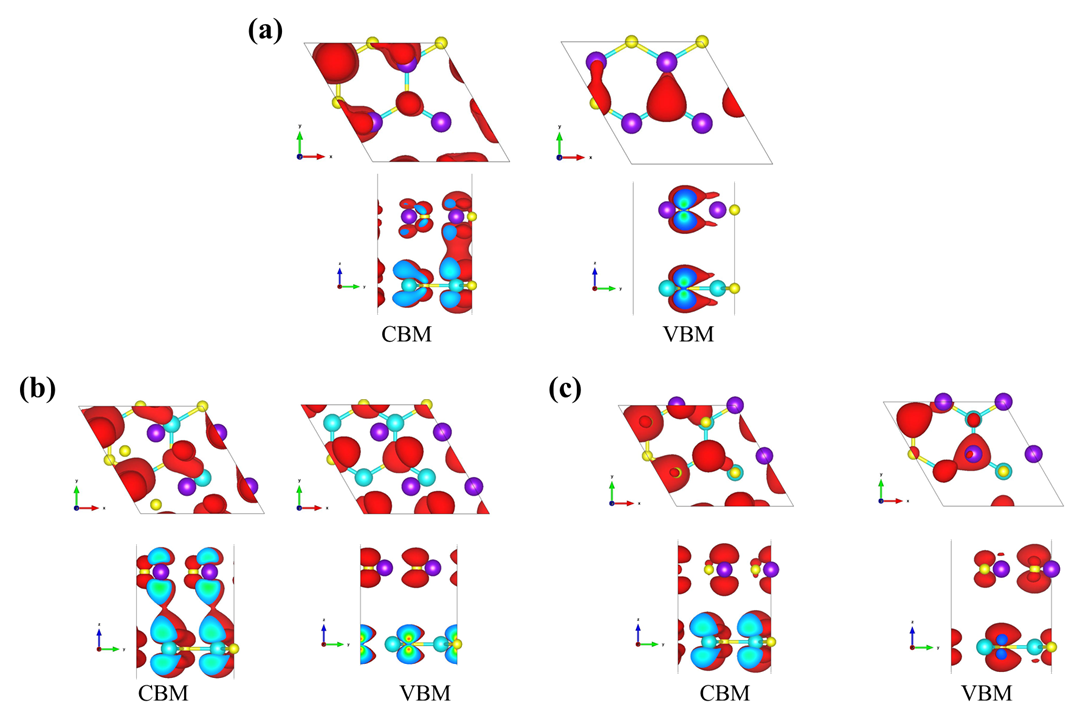
Figure S2**. Charge density distribution at the VBM and CBM (top view and side view) of (a) Structure I, (b) Structure II, and (c) Structure III of 2D GeC/SiC heterobilayer. The iso value is 0.0015 ${eÅ}^{-3}$. The violet, cyan, and yellow color refers to Ge, Si, and C atoms, respectively. The red color indicates the density of charge distribution.

The charge redistribution in VBM and CBM upon application of biaxial strain is shown in Fig. S3. At zero strain, the Ge and Si atoms of structures I and II are hybridized in the CBM, which improves charge transport between the GeC and SiC layers. However, even though the charge appears predominantly near the Ge and Si atoms in structure III, they are not hybridized. The Ge and Si atoms in structure I remain hybridized when tensile strain is incorporated (at +2% strain). Charge transfer takes place between the C and Si atoms of the SiC layer in structure II. When structure III is strained, the charge near the Ge atoms increases slightly. The interlayer charge transfer between the Si-C and Ge-C atoms increases when compressive strain is applied to any of the structures. The change of VBM is quite negligible, except for the fact that charge accumulation near the C atoms slightly decreases with tensile strain. Charge redistribution among the Ge, Si, and C atoms in the GeC and SiC layers will cause an electrostatic potential shift from the SiC to the GeC layer, thereby altering the active reaction sites at the GeC and SiC surface to carry out the water-splitting process using photogenerated electrons and holes.


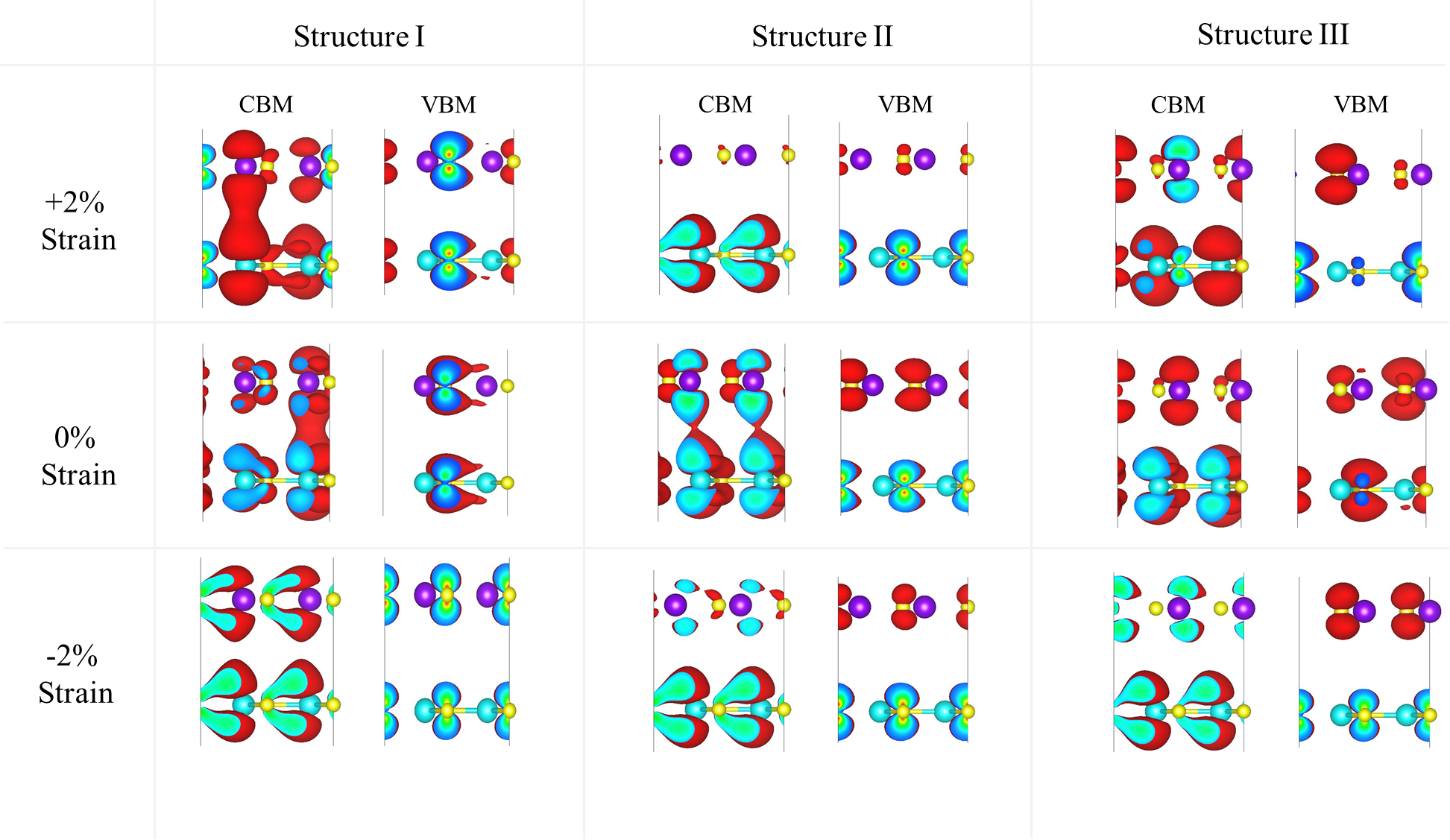


**Figure S3.** Change of charge distribution at the valence and conduction bands when external strain is incorporated into the GeC/SiC vdW heterobilayers. The iso value is 0.0015 ${eÅ}^{-3}$. The violet, cyan, and yellow color refers to Ge, Si, and C atoms, respectively. The red color indicates the density of charge distribution.
